# Estimating the total treatment effect in randomized experiments with unknown network structure

**DOI:** 10.1073/pnas.2208975119

**Published:** 2022-10-24

**Authors:** Christina Lee Yu, Edoardo M. Airoldi, Christian Borgs, Jennifer T. Chayes

**Affiliations:** ^a^School of Operations Research and Information Engineering, Cornell University, Ithaca, NY 14850;; ^b^Department of Statistics, Operations, and Data Science, Fox School of Business, Temple University, Philadelphia, PA 19122;; ^c^Department of Electrical Engineering and Computer Science, University of California, Berkeley, CA 94720;; ^d^Department of Electrical Engineering and Computer Science, University of California, Berkeley, CA 94720;; ^e^Department of Statistics, University of California, Berkeley, CA 94720;; ^f^Department of Mathematics, University of California, Berkeley, CA 94720;; ^g^School of Information, University of California, Berkeley, CA 94720

**Keywords:** design of experiment, additive network interference, heterogeneous causal effects, social and information networks, total treatment effect

## Abstract

Randomized experiments are widely used to estimate the causal effects of a proposed treatment in many areas of science, from medicine and healthcare to the physical and biological sciences, from the social sciences to engineering, and from public policy to the technology industry. Here we consider situations where classical methods for estimating the total treatment effect on a target population are considerably biased due to confounding network effects, i.e., the fact that the treatment of an individual may impact its neighbors’ outcomes, an issue referred to as network interference or as nonindividualized treatment response. A key challenge in these situations is that the network is often unknown and difficult or costly to measure. We assume a potential outcomes model with heterogeneous additive network effects, encompassing a broad class of network interference sources, including spillover, peer effects, and contagion. First, we characterize the limitations in estimating the total treatment effect without knowledge of the network that drives interference. By contrast, we subsequently develop a simple estimator and efficient randomized design that outputs an unbiased estimate with low variance in situations where one is given access to average historical baseline measurements prior to the experiment. Our solution does not require knowledge of the underlying network structure, and it comes with statistical guarantees for a broad class of models. Due to their ease of interpretation and implementation, and their theoretical guarantees, we believe our results will have significant impact on the design of randomized experiments.

## Significance

In many domains, we want to estimate the total treatment effect (TTE) in situations where we suspect network interference is present. However, we often cannot measure the network or the implied dependency structure. Surprisingly, we are able to develop principles for designing randomized experiments without knowledge of the network, showing that under reasonable conditions one can nonetheless estimate the TTE, accounting for interference on the unknown network. The proposed design principles, and related estimator, work with a broad class of outcome models. Our estimator has low variance under simple randomized designs, resulting in an efficient and practical solution for estimating total treatment effect in the presence of complex network effects. We detail the assumptions under which the proposed methods work and discuss situations when they may fail.

The measurement of treatment effects via randomized experiments is a fundamental tool used across all fields of scientific disciplines and beyond. For example, consider a public health campaign to increase public awareness of the importance of wearing masks during a global pandemic. The administrator in charge of running the public health campaign wants to determine which proposed banner ad would be most effective for displaying on a public billboard. In particular, the administrator wants to estimate the total treatment effect, i.e., the change in behavior of the population at large that results from viewing the proposed banner ad. The total treatment effect is a causal effect, as it describes the change in behavior that is caused by the treatment. The experimental units in this example are the individuals in the population, and outcomes refer to some measurable behavior of individuals, such as whether an individual is wearing a mask or not at the grocery store.

The classical approach to estimating causal effects involves running a randomized experiment, where one randomly partitions the population into a treatment group and a control group. The treatment of interest is administered to each individual in the treatment group, and a placebo is administered to each individual in the control group. The causal effect is then approximated by the difference in measured outcomes or behaviors between the treatment and control groups after the treatment has been administered. This approach results in an efficient unbiased estimate for the desired causal effect under a critical assumption that the outcome of an individual is not affected by the treatment assignment of any other individual; this assumption is referred to in the literature as the stable unit treatment value assumption (SUTVA) (1–3).

Unfortunately, SUTVA is violated in many applications, as individuals are connected in a complex social network that mediates communication, influence, or spread of disease, resulting in network interference that couples the outcomes of individuals. The treatment of individual A may impact the outcome of individual B, violating SUTVA and introducing significant bias to the estimates resulting from the classical experimental approach of randomizing treatment and control uniformly over individuals and estimating the difference in average outcomes of the treatment and control group. Furthermore, the network that mediates the interference effects is often unknown and difficult or costly to estimate. As a result, we need a theory for experimental design that can account for these network interference effects in the absence of observations about the network, and yet is simple and practical to implement. We illustrate a few motivating scenarios.

Consider estimating the total treatment effect of a public health campaign to increase the use of masks in public during a pandemic. Suppose that individual A, a senior citizen, is shown the proposed banner ad and thus decides to wear a mask in public. Individual A’s behavior could cause a positive network effect on the individual’s friends, even though they did not see the original banner ad. In contrast, individual A’s behavior may have a negative effect on a teenager, who may think that wearing masks must be for the elderly and thus not “cool.”

Consider a social media platform such as LinkedIn, which wants to estimate the total treatment effect of a proposed change in the recommendation engine for a user’s news feed, with a goal of increasing user engagement. The change in engagement level of individual A as a result of being exposed to the proposed change could subsequently impact the engagement level of others in individual A’s social network, resulting in a positive or negative network effect. Similar network effects arise in other types of communication networks beyond social media platforms, including mobile networks, email exchange networks, and collaboration communities.

Consider running a clinical trial to estimate the total treatment effect of a proposed vaccine for COVID-19, i.e., how much the overall rate of cases contracted in the public at large would decrease as a result of everyone receiving the vaccine. Since COVID-19 is transmitted via an underlying social contact network, the impact of individual A receiving the vaccine may not only reduce individual A’s chance of contracting the disease, but also reduce the risk of exposure of other individuals connected to A in the network. This network effect is heterogeneous as the frequency of time individual A spends with others in individual A’s contact network may vary.

## Problem Setup and Potential Outcomes Model

Consider a finite population of *n* individuals. We denote the treatment vector by $z=(z_{1},z_{2},\ldots z_{n})\in {\left\{0,1\right\}}^{\left[n\right]}$, where $z_{i}=1$ if individual *i* is assigned the treatment and $z_{i}=0$ if individual *i* is in the control group. Let $e_{i}$ denote the standard basis vector that takes value one at coordinate *i* and is zero everywhere else. As we consider the randomized experiment setting, we assume that the treatment vector **z** is sampled from a prescribed distribution as determined by the experimental design; this distribution is referred to as the randomized design. In many practical applications, there is a limit on the fraction of individuals that can be assigned to the treatment group, whether because of a high cost for testing a new treatment or due to safety considerations of limiting possible unknown adverse effects. Therefore, a desired solution involves proposing an estimator alongside a randomized design for which we can achieve consistent estimation while keeping the expected number of treated individuals low.

**Fig. 1.**
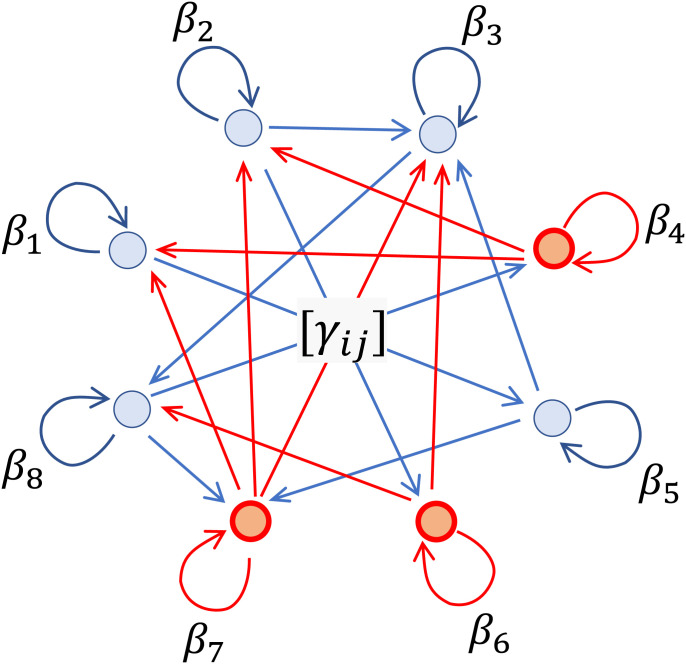
Depiction of model under heterogeneous additive network effects: Vertex weights correspond to baseline outcomes. When individual *i* is treated (depicted in red), individual *i*’s outgoing edges are activated. Individual *j*’s outcome $Y_{j}(z)$ is the sum of individual *j*’s own baseline plus any incoming activated edges (depicted in red).

$Y_{i}(z)$ denotes the potential outcome of individual *i* in the event that treatment vector **z** is implemented. Only the outcomes for the implemented treatment vector **z** are observed, and thus all other “potential outcomes” that would result from other realizations of the treatment vector are unobserved. Under the stable unit treatment value assumption, the potential outcome of individual *i* depends only on *z_i_* and not on the treatment of any other individual (1–3). Under this assumption, $Y_{i}(z)=Y_{i}(z_{i}e_{i})$ for all **z**. In the presence of general arbitrary network interference, the outcome of individual *i* may depend on the full treatment vector. Our results rely on the neighborhood interference assumption alongside joint assumptions of additivity of main effects and interference effects as defined in ref. 4, which we also refer to as the heterogeneous additive network effects assumption (Fig. 1).

### Heterogeneous Additive Network Effects.

The neighborhood interference assumption posits that an individual’s outcome can only depend on the treatment assignments of its direct neighbors in a specified static network E (4, 5). We furthermore assume that the network is unknown. The joint assumptions of additivity of main effects and interference effects as defined in ref. 4 impose that the potential outcomes satisfy

$$Y_{i}(z)\text{​}=\text{​}Y_{i}(0)\;+\;(Y_{i}(e_{i})\text{​}-\text{​}Y_{i}(0))\;+\;\sum_{k\in [n]}(Y_{i}(e_{k})\text{​}-\text{​}Y_{i}(0)).$$

This enforces that the outcome of each individual is affected by an additive term for each treated individual in the population, but this additive network interference effect can be fully personalized to each pair of individuals. By letting *α_i_* denote the individual baseline outcome $Y_{i}(0)$, *β_i_* denote the individual direct effect $\left(Y_{i}(e_{i})-Y_{i}(0)\right)$, and *γ_ki_* denote the additive network interference effect over the directed pair (*k*, *i*) given by $\left(Y_{i}(e_{k})-Y_{i}(0)\right)$, it follows that the potential outcomes model is equivalently represented as

$$Y_{i}(z)=\alpha_{i}+\beta_{i}z_{i}+\sum_{k\in [n]}\gamma_{ki}z_{k}.$$

This model trivially satisfies the neighborhood interference assumption with respect to the network edge set E defined as the set of pairs (*k*, *i*), where $\gamma_{ki}\neq 0$. The total number of model parameters is 2n+|E|. Without imposing constraints on the sparsity of E, |E| could be as large as n(n−1).

The model at a glance looks similar to a linear model, yet a key distinction is that our model allows for fully heterogeneous network effects individualized to each edge, such that the number of parameters grows with the population size. This model is also referred to as the linear model in ref. 6 or the saturated structural linear model in ref. 7. Our model is significantly more expressive than typical linear models used in the empirical literature that impose that there are a fixed number of types of individuals that then share the same network effect coefficients, such that the number of model parameters is fixed with respect to the population size.

### Target Estimand.

There are many potential estimands of interest; we focus on the total treatment effect (TTE), defined as the difference between the average outcome if all individuals were treated and the average outcome if nobody were treated:

$$\text{TTE}≔\frac{1}{n}\sum_{i\in [n]}(Y_{i}(1)-Y_{i}(0)),$$

where 1 denotes the vector of all ones and 0 denotes the vector of all zeros. Under heterogeneous additive network effects, the TTE estimand takes value

$$\text{TTE}=\frac{1}{n}\left(\sum_{i}\beta_{i}+\sum_{ki}\gamma_{ki}\right)\text{​}.$$

The total treatment effect is particularly relevant in scenarios in which a decision maker can run a randomized experiment with a limited treatment budget and desires to use the outcome of the experiment to determine whether to fully adopt the new treatment or to stay with the status quo. The challenge is that the decision maker wants to estimate the outcomes under the all ones treatment vector, but due to a limited budget, the decision maker can only observe outcomes under a limited treatment budget. The total treatment effect can be decomposed into a direct effect, a network interference effect, and an interaction effect (4). The direct effect captures the change in outcomes of an average individual due to the individual being treated. The network interference effect captures the change in outcomes of an average individual due to the network (excluding the individual) being treated. The interaction effect is nonzero in scenarios when the effect of interference on an individual may depend on whether the individual is treated or not.

Some previous work has focused on estimating the direct effect (4, 8–12) or testing for the presence of network interference (13–17); these methods do not produce an estimate of the total treatment effect. The techniques for hypothesis testing in refs. 13 and 15 do not immediately extend to estimation as they are based on randomization inference with a fixed network size and studied testing sharp null hypotheses. While this paper focuses on estimating the total treatment effect, we show results for the direct treatment effect and the network interference effect in *SI Appendix*.

### Class of Estimators.

A primary question of this work is to understand whether one can estimate total treatment effect in the presence of network interference, particularly when the network structure is unknown and costly to estimate, which is often the case in many real world applications. As a result, we consider the following class of individually weighted linear estimators, which have the form

$$\hat{\text{est}}(w,v)=\sum_{i\in [n]}\left(w_{i}z_{i}+v_{i}(1-z_{i})\right)Y_{i}(z),$$

where the weights $w=(w_{1},w_{2},\ldots w_{n})$ and $v=(v_{1},v_{2},\ldots v_{n})$ are deterministic and not a function of the treatment **z**. Most notably, the weight *w_i_* or *v_i_* is selected only based on whether individual *i* is treated or not and does not depend on the treatment configuration of its neighbors.

The focus on linear estimators is not restrictive, as the majority of all estimators proposed in the literature are indeed linear in the measured outcomes. However, the limitation that the weights that multiply an individual’s outcome $Y_{i}(z)$ depend only on that individual’s treatment *z_i_* is a significant restriction and arises from the limitation that we do not have knowledge of the network. In contrast, the Horvitz–Thompson estimator under general neighborhood interference is a linear estimator where the weight that multiplies $Y_{i}(z)$ is a function of the treatments of all neighbors of *i* in addition to *i* itself; this necessarily requires knowledge of the neighborhood (5). All estimators previously studied in the network interference literature require some knowledge of the network.

Under the simplifying SUTVA condition, many classic estimators are in fact individually weighted linear estimators. For example, the Horvitz–Thompson estimator under SUTVA sets $w_{i}=1/(nE[z_{i}])$ and $v_{i}=1/(nE[1-z_{i}])$. The difference in means estimator sets $w_{i}=1/\sum_{j\in n}z_{j}$ and $v_{i}=1/\sum_{j\in n}\left(1-z_{j}\right)$, which are deterministic for randomized designs in which the total numbers of individuals under treatment and control are fixed.

### Discussion of Model Assumptions.

Our model assumes a finite population of size *n* with arbitrary values for $\alpha_{i},\beta_{i},\gamma_{ki}$. Since the model allows for any abitrary values, it also can capture a model in which these unknown parameters are generated from an underlying stochastic process, with respect to which the parameters across individuals could be correlated. For example, in an application such as estimating the efficacy of a vaccine, an underlying network mediates the spread of the epidemic under both control and treatment scenarios. If ${\left\{s_{i}\right\}}_{i\in [n]}$ describes the initial seeds of an infection, and *T_ji_* describes the accumulated transmission (potentially over multiple hops of the network) from a seed *j* to individual *i*, then the baseline outcomes would take the form of $\alpha_{i}=\sum_{j\in [n]}T_{ji}s_{j}$. The baseline parameters *α_i_* are thus correlated with respect to the shared dependence on the random initial seeds *s_j_*, but as the baseline parameters are assumed to be indepedent from the treatment assignments, our results will still hold. In particular, our analysis considers the randomness of the outcomes with respect to the treatment vector **z** conditioned on the realized baseline parameters *α_i_*, treating them as constant.

This model can also capture network interference that arises from spillover, peer effects, and contagion. Spillover refers to the interference that arises from individual *j*’s treatment affecting individual *i*’s outcome. Typically the spillover effect is assumed to be mediated by the network, such that *γ_ji_* is nonzero only if (*j*, *i*) is an edge in the network. By relaxing constraints on the sparsity of the *γ_ki_* parameters, the heterogeneous additive network effects assumption can also capture long-range spillover effects mediated by multihop paths in the network.

Contagion or peer effects refer to interference that arises from individual *j*’s outcome affecting individual *i*’s outcome. When the contagion effect is linear, then this translates into long-range interference effects over multihop paths in the network. For example, consider a path ℓ→k→j→i in the network. Under linear contagion, individual ℓ’s treatment affects individual ℓ’s outcome, which subsequently affects individual *k*’s outcome, which subsequently affects individual *j*’s outcome, which then affects individual *i*’s outcome, as described by

$$Y_{i}(z)=a_{i}+b_{i}z_{i}+\sum_{k\in [n]}c_{ki}Y_{k}(z).$$

The potential outcomes model can be derived by solving a system of linear equations for the outcomes vector given an assigned treatment vector, which results in the following potential outcomes model:

$$Y(z)={\left(I-C\right)}^{-1}a+\sum_{t=0}^{\infty }C^{t}\cdot \text{diag}(b)\cdot z,$$

where *C* is a matrix with the (*k*, *i*)th entry equal to *c_ki_*, diag(b) is a diagonal matrix with diagonal entries taking values from **b**, and **a** and **b** are vectors corresponding to the parameters ${\left\{a_{i}\right\}}_{i\in [n]}$ and ${\left\{b_{i}\right\}}_{i\in [n]}$. Written in this form, we can verify that the potential outcomes could be described via a heterogeneous additive network effects model with dense network effects, as is also observed in refs. 6 and 7.

As there are more unknown model parameters than measurements, we cannot hope to identify the model via regression, and thus a randomized experimental design will be critical to any solution. Previous attempts at causal inference under this model involve complicated network-dependent randomized designs (6), incur potentially high network-dependent biases (6), or impose Bayesian priors on the unknown parameters that reduce the statistical estimation task to again estimating a model with a fixed number of parameters (8, 18).

Scenarios that violate linearity include when network effects saturate after a certain number of neighbors are treated, are sublinear in the number of treated neighbors, or are present only after a minimum number of neighbors are treated. Linearity is naturally violated if the measured outcome variable is binary valued. As a result, our model is more suited to settings where *Y_i_* takes a spectrum of values, such as representing the viral load an individual has accumulated rather than the individual’s binary infection status.

## Alternate Approaches in the Literature

A critical challenge for estimating the total treatment effect is that we observe ${\left\{Y_{i}(z)\right\}}_{i\in [n]}$ only for a single fixed treatment vector **z**, which is not 1 or 0. As a result, we may not observe any of the terms in the expression of interest. Under a fully general arbitrary interference model, it has been repeatedly shown that it is impossible to estimate any desired causal estimands as the model is not fully identifiable (3, 19–21). As a result, there have been many proposed models that impose assumptions on exposure functions (3, 19, 22–24), interference neighborhoods (4, 5, 25, 26), parametric structure (6, 8, 18, 27, 28), or a combination of these. Each of these assumptions leads to a different solution concept. The art in choosing a good model is balancing the tension between strong assumptions that facilitate simple solutions and weak assumptions that can more flexibly encompass real world applications. Furthermore, studies show that one must exercise caution in choosing model assumptions, as the results may be sensitive to model misspecification (19, 21).

All previously proposed approaches critically rely on using knowledge of a network mediating the interference effects, which is often not available in practice. We highlight a few of the most common models to highlight the strengths and weaknesses of each approach. In complement to the below works, there are ongoing empirical studies assessing the performance difference between an experiment design that leverages the network implicitly and a method that measures the network and leverages the measured network (29).

### Partial Interference.

Partial interference assumes that the population can be partitioned into disjoint groups, such that all network interference effects can only occur within but not across the prespecified groups (23, 26, 30–35). Specifically, the outcome of individual *i* can only depend on the treatment of others in the same group as individual *i* and is independent from the treatment assignments of individuals in other groups. Under this assumption, we can randomize treatments over the groups jointly so that all individuals in each group are assigned jointly either to treatment or to control. As a result, $Y_{i}(z)=Y_{i}(1)$ for all *i* such that $z_{i}=1$, and $Y_{i}(z)=Y_{i}(0)$ for all *i* such that *z_i_* = 0. Unfortunately, this approach does not apply when the network could be highly connected, limiting its use in practice. The bias of standard estimators will scale with the number of edges across clusters, leading to proposed cluster randomized designs that randomize over clusters that are constructed to minimize edges between clusters (6, 28).

### Neighborhood Interference.

Under the neighborhood interference assumption, $Y_{i}(z)=Y_{i}(1)$ for any individual in the treatment group whose neighbors are also all in the treatment group; we denote this set of individuals $S_{1}(z)$. Similarly, $Y_{i}(z)=Y_{i}(0)$ for any individual in the control group whose neighbors are also all in the control group; we denote this set $S_{0}(z)$. Without imposing any further assumptions, a natural estimate for the total treatment effect is the difference in average outcomes between groups $S_{1}(z)$ and $S_{0}(z)$ or an inverse probability weighted estimator when the probability of being in group $S_{1}(z)$ or $S_{0}(z)$ may vary across individuals (19). Without further structure on the interference, one cannot use measurements from individuals not in either set $S_{1}(z)$ or set $S_{0}(z)$, as the relationship between $Y_{i}(z)$ and $Y_{i}(1)$ or $Y_{i}(0)$ is unknown.

Under naive randomized designs such as a Bernoulli design that assigns each individual independently to treatment with probability *p* or control with probability 1−p, the variance of the inverse probability weighted estimator will go to infinity with *n* for well-connected networks such as the Erdos–Renyi graph with average degree larger than $\sqrt{n}$ (20); this results from the fact that with high probability the sizes of sets $S_{1}(z)$ and $S_{0}(z)$ will be small for highly connected networks. As a result, ref. 5 proposes a graph cluster randomized design that aims to jointly assign individuals and their neighbors to treatment or control to minimize variance. Unfortunately this requires detailed knowledge of the network, and constructing optimal clusters can be computationally expensive for nontrivial well-connected networks. As a result, this approach has not translated into practical solutions.

An alternate approach suggested by refs. 7 and 36 is to instead consider weaker estimands, which essentially capture the marginal treatment effect of perturbing a status quo treatment assignment distribution. To show a central limit theorem-styled result, ref. 36 imposes a generative distribution over the network structure itself and considers how to exploit the regularity in the network that arises from low rank structure. While this enables one to consider networks of increasing size, it may only be plausible in applications in which one can reasonably model the finite network as being sampled from a known generative model.

### Linear Model with Fixed Number of Parameters.

While the above models impose network-based conditions on the interference, an alternate approach is to impose parametric structure on the form of the potential outcomes. The most common assumption is that the potential outcomes are linear with respect to a specified statistic of the local neighborhood (8, 18, 27, 28, 37, 38). For example, ref. 27 assumes the outcome is linear in the fraction of treated neighbors, such that

$$Y_{i}(z)=\alpha +\beta z_{i}+\gamma \left(\frac{\sum_{k\in [n]}A_{ki}z_{k}}{\sum_{k\in [n]}A_{ki}}\right)\text{​},$$

where *A* is a known adjacency matrix representing edges in the network. Similarly, ref. 37 assumes linearity with respect to the absolute number of treated neighbors. Threshold models also can be expressed with a linear model using indicator statistics. For example, ref. 28 assumes that network effects arise when at least *θ* neighbors are treated, where *θ* is assumed to be known,

$$Y_{i}(z)=\alpha +\beta z_{i}+\gamma \;I(\sum_{k\in [n]}A_{ki}z_{k}\geq \theta ).$$

One can extend these models to incorporate covariate types, such that the total number of unknown parameters is three times the number of different covariate types, assuming that each covariate type is associated to a set of parameters *α*, *β*, *γ*.

What is characteristic of this approach is that the assumptions reduce the number of unknown parameters in the potential outcomes models to a fixed dimension that does not grow with the population size, reducing the inference task to linear regression. As a result, the natural solution is to use a least-squares estimate, shifting the focus to constructing randomized designs that minimize the variance of the estimate. A limitation of this approach is that it requires the correct choice of the the statistic governing the linearity, and it requires precise knowledge of the network structure to compute these neighborhood statistics. Furthermore, it assumes knowledge of the relevant covariate types that differentiate individual responses or otherwise assumes homogeneity in the effects.

## Summary of Our Results

Our results focus on estimating the total treatment effect without knowledge of the network under a heterogeneous additive network effects model, which is significantly more expressive than parametric model classes, where the number of parameters does not scale with the population size. Under our model, the total treatment effect scales linearly in the fraction of treated individuals; our approach exploits this linearity for a simple and efficient solution. Our results offer methods and theory that do not require knowledge of the network. We believe this combination of a practical solution with a flexible model positions our results to have impact in the broader scientific community.

The primary research question is, Does there exist a simple and efficient solution for estimating total treatment effect in the presence of network effects without critically relying on knowledge of the network structure or restrictive network properties? While this has previously remained elusive, our results provide a clear and simple answer, including both a negative scenario in which there can be no simple solution and a positive scenario in which we outline a simple efficient solution that can easily translate into practice.

First, we show that in the presence of additive network effects, any individually weighted linear estimator for the TTE is necessarily biased unless the network can be perfectly partitioned into small disjoint subsets with no interfering edges. Furthermore, this bias can be large, depending on the relative magnitude of the network effects. This negative result suggests that the linearity arising from the additive network effects model is not sufficient in itself to admit simple estimators that do not utilize knowledge of network structure. The primary reason is that it is difficult for simple estimators to distinguish between the response due to baseline values ${\left\{\alpha_{i}\right\}}_{i\in [n]}$ and that due to network effects ${\left\{\gamma_{ji}\right\}}_{(j,i)\in E}$.

Second, we consider the scenario when we have access to an estimate of the average individual baselines; in practice this could be constructed from historical data or pilot studies. Given baseline estimates, we propose a simple estimator for the total treatment effect that computes the average outcomes among the entire population after applying the treatment vector, scales the average outcome by the size of the total population divided by the number of treated individuals, and then subtracts the average baseline estimate. This estimator is unbiased for any randomized design in which the marginal probability of an individual being treated is equal among different individuals in the population, an easy condition to satisfy as it involves only matching individual marginal treatment probabilities. This estimator is extremely easy to compute, and neither the randomized designs nor the estimate itself require knowledge of the underlying network, which is often not available in practice.

Third, we show that our proposed approach has low variance under a simple completely randomized design. In particular, the estimator is consistent as long as the fraction of treated individuals is asymptotically larger than $d_{\max}^{2}/n$, where $d_{\max}$ is the maximum outdegree of any individual in the network, i.e., the maximum influence of any individual in the network. Furthermore, we provide analytical expression for the variance of our estimator under commonly used randomized designs, including completely randomized and cluster-randomized design, as well as uniform and varying saturation designs. These variance expressions provide insight for designing randomized designs that minimize variance by matching individuals based on estimated network influence.

## Additivity Is Not Sufficient for Unbiased Estimators

Assuming heterogeneous additive network effects implies that the total treatment effect scales linearly in the number of treated individuals. A natural question is whether this additive model is sufficient to admit simple unbiased estimators for the total treatment effect or not. In this section we provide some results in the negative by considering the restricted class of individually weighted linear estimators.

Theorem 1. *Under heterogeneous additive network effects*, *any unbiased individually weighted linear estimator for total treatment effect must have the form*

$$\hat{\text{TTE}}=\frac{1}{n}\sum_{i\in [n]}\left(\frac{z_{i}}{E[z_{i}]}-\frac{1-z_{i}}{E[1-z_{i}]}\right)Y_{i}(z),$$

*and the randomized design must satisfy* $\mathbb{P}(z_{k}=z_{i})=1$ *for all* (k,i)∈E*. As a result*, *there does not exist an unbiased individually weighted linear estimator for the total treatment effect if the network is fully connected.*

Proof.
Under the heterogeneous additive network effects model, an individually weighted linear estimator takes the value

$$\begin{array}{l} \hat{\text{est}}(w,v)=\sum_{i\in [n]}(w_{i}z_{i}+v_{i}(1-z_{i}))\alpha_{i}+\sum_{i\in [n]}w_{i}z_{i}\beta_{i}\\ \;+\sum_{(k,i)\in E}(w_{i}z_{i}+v_{i}(1-z_{i}))z_{k}\gamma_{ki}. \end{array}$$

This is an unbiased estimator for the total treatment effect only if $E[\hat{\text{est}}(w,v)]=\frac{1}{n}\sum_{i\in [n]}\beta_{i}+\frac{1}{n}\sum_{(k,i)\in E}\gamma_{ki}$ is satisfied for any configuration of ${\left\{\alpha_{i}\right\}}_{i\in [n]},{\left\{\beta_{i}\right\}}_{i\in [n]}$, and ${\left\{\gamma_{ki}\right\}}_{(k,i)\in E}$. This requirement results in the following 2n+|E| constraints, which arise from matching coefficients for each of the parameters:

• *α*: For all $i\in [n],\;w_{i}E[z_{i}]+v_{i}E[1-z_{i}]=0$.
• *β*: For all $i\in [n],\;w_{i}E[z_{i}]=\frac{1}{n}$.
• *γ*: For all $(k,i)\in E,\;w_{i}E[z_{i}z_{k}]+v_{i}E[(1-z_{i})z_{k}]=\frac{1}{n}$.

The second set of constraints for the direct treatment effects implies that the weights are $w_{i}=1/(nE[z_{i}])$. As a result, combining this with the first set of constraints for the baselines implies that $v_{i}=-1/(nE[1-z_{i}])$. After fixing the values of all the weights w,v, the third set of constraints becomes difficult to satisfy. We can rewrite the third set of constraints as

$$w_{i}E[z_{i}]\mathbb{P}(z_{k}=1|z_{i}=1)+v_{i}E[1-z_{i}]\mathbb{P}(z_{k}=1|z_{i}=0)=\frac{1}{n},$$

for all (k,i)∈E. Most notably, it is a linear combination of the two terms that shows up in the first and second sets of constraints, multiplied by probabilities that must be in [0,1]. By plugging in the values for *w_i_* and *v_i_* that arise from the first two constraints, it follows that the third constraint is satisfied only when $\mathbb{P}(z_{k}=1|z_{i}=1)=1$ and $\mathbb{P}(z_{k}=1|z_{i}=0)=0$ for all (k,i)∈E. This is equivalent to requiring that the randomized design always assigns connected individuals to the same treatment; i.e., $\mathbb{P}(z_{k}=z_{i})=1$ for all (k,i)∈E.□

The constraint on the randomized design implies that every pair of connected individuals in the population must be either both treated or both control. This restricts the valid randomized designs to a cluster-randomized design where the clusters are defined by the connected components of the graph. *Theorem 1* highlights that the imposed structure from heterogeneous additive network effects is insufficient to remove the complex dependence on the network. Even under linearity, we still need to deal with imposing strong assumptions on the connectivity structure of the network, or we will need to use more complex estimators that utilize knowledge of the network, bringing us back to the same challenges present in the fully general model.

When the conditions for unbiasedness are not satisfied, the bias of the above simple estimator will scale with the average network effect across the edges between the treated and control groups, given by the expression

$$E[\hat{\text{TTE}}]-\text{TTE}=\frac{1}{n}\sum_{(k,i)\in E}\left(\frac{\text{Cov}[z_{i},z_{k}]}{\text{Var}[z_{i}]}-1\right)\gamma_{ki}.$$

If the randomized design produces high correlation in treatments across pairs of connected individuals in the network, then $E[\hat{\text{TTE}}]$ is close in expectation to the total treatment effect. If the design enforces independence of treatments across edges in the network, then $E[\hat{\text{TTE}}]$ captures only the direct treatment effects and not the network effects.

The restrictive unbiasedness conditions result from the fact that it is difficult to set the coefficient for the baseline parameters to 0 while maintaining that the coefficients on the network effects are 1/n, as the expressions for both are very similar. Essentially, it is difficult for the model to distinguish between the effects arising from individual baselines and the ambient network effects from treated neighbors. Given this insight, in the next section we consider the scenario where we have access to estimates of the average individual baselines.

## Simple Unbiased Estimator Given Baseline Estimates without Any Knowledge of the Network

In practice there are many applications in which we do have access to additional information from historical data or pilot studies that could be used to construct estimates of the average baseline $\frac{1}{n}\sum_{i\in [n]}Y_{i}(0)=\frac{1}{n}\sum_{i\in [n]}\alpha_{i}$. For example, a social media platform such as LinkedIn is constantly monitoring the engagement level of its users, such that it always has access to the current status quo baselines at an individual level before deploying randomized trials for a newly proposed feature. Even when historical data may not be available, it is typically easy to conduct small-scale surveys to estimate the baseline outcome levels before beginning the randomized experiment. The data must be collected before the experiment begins such that no one has yet received the treatment. Under our heterogenous additive network effects assumption, the measurements collected before the experiment will accurately reflect the baseline with no network effects due to treatment; these additional data can then be used to significantly simplify the estimation of causal effects.

Let us first assume that we have access to the full individual baselines; it follows naturally to then subtract the baseline *α_i_* from the measurement $Y_{i}(z)$ to remove all contributions of the baseline effects from the linear estimator, resulting in

$${\hat{\text{est}}}_{-\alpha }(w,v)=\sum_{i\in [n]}\left(w_{i}z_{i}+v_{i}(1-z_{i})\right)(Y_{i}(z)-\alpha_{i}).$$

To characterize conditions for unbiased linear estimators, we use the same approach of equating the coefficients of the direct effects and the network effects between the expected value of this estimator and the total treatment effect. Subtracting out the baselines removes the set of constraints for unbiasedness associated to the baseline parameters, leaving us with n+|E| constraints. While this is still significantly more than the number of measurements, it turns out that there are still many reasonable randomized designs under which we are able to satisfy these constraints. *Theorem 2* presents sufficient conditions for unbiased linear estimators for total treatment effect given baseline estimates.

Theorem 2. *Under heterogeneous additive network effects*, *for any randomized design such that* $\frac{\mathbb{P}(z_{k}=0\;|\;z_{i}=1)}{\mathbb{P}(z_{k}=1\;|\;z_{i}=0)}=\rho_{i}$ *for all* (k,i)∈E *for some values of* ${\left\{\rho_{i}\right\}}_{i\in [n]}$, *the following estimator*,

$${\hat{\text{TTE}}}_{-\alpha }=\frac{1}{n}\sum_{i\in [n]}\left(\frac{z_{i}}{E[z_{i}]}-\frac{(1-z_{i})\rho_{i}}{E[1-z_{i}]}\right)(Y_{i}(z)-\alpha_{i}),$$

*produces an unbiased estimate for the total treatment effect.*

The condition on the randomization is equivalent to imposing that

$$\frac{E[z_{i}(1-z_{k})]}{E[(1-z_{i})z_{k}]}=\frac{E[z_{i}(1-z_{j})]}{E[(1-z_{i})z_{j}]}$$

for all triplets (*i*, *j*, *k*) such that (k,i)∈E and (j,i)∈E. Essentially this boils down to symmetry conditions on the second moments of the treatment vector across edges in the network. Such a symmetry condition would be satisfied by ensuring that for all *i*, the neighbors that influence *i* are treated equally in the distribution of the assigned treatments. The ratio above can be expanded to

$$\frac{E[z_{i}(1-z_{k})]}{E[(1-z_{i})z_{k}]}=\frac{E[z_{i}]-E[z_{i}z_{k}]}{E[z_{k}]-E[z_{i}z_{k}]},$$

which is equal to 1 if $E[z_{i}]=E[z_{k}]$. As a result, a sufficient condition to satisfy the required symmetry is to impose that the marginals are equal across edges; i.e., $E[z_{i}]=E[z_{k}]$ for all (k,i)∈E. This is an easy condition to satisfy and leads to a simplified result as stated below.

Corollary 3. For any randomized design such that $E[z_{i}]=E[z_{k}]$ for all (k,i)∈E, the following simple estimator

$${\hat{\text{TTE}}}_{-\alpha }=\frac{1}{n}\sum_{i\in [n]}\frac{Y_{i}(z)-\alpha_{i}}{E[z_{i}]}$$

*produces an unbiased estimate for the total treatment effect under heterogeneous additive network effects. When* $E[z_{i}]=p$ *for all* i∈[n], *the estimator further simplifies to*

$${\hat{\text{TTE}}}_{-\alpha }=\frac{1}{p}\left(\frac{1}{n}\sum_{i\in [n]}Y_{i}(z)-\frac{1}{n}\sum_{i\in [n]}\alpha_{i}\right)\text{​}.$$

It may seem that we need knowledge of the network to choose a distribution satisfying the symmetry conditions, as they are defined with respect to constraints over the edges; however, without knowledge of the network, one could use a distribution with uniform marginal treatment probabilities as it satisfies the required conditions for even the complete graph, which is most restrictive. When the marginal treatment probability is equal for all individuals, i.e., $E[z_{i}]=p$ for all i∈[n], the resulting estimator in fact needs only knowledge of the average population baselines rather than individual baseline parameters. While there are a few settings for which individual baseline parameters are observed from historical data, such as experimentation on social media platforms, data of such granularity are not realistic in general. On the other hand, having access to an accurate estimate of the average population-wide baselines is realistic for a broad variety of applications across public health and social sciences, as the population-wide statistic could be estimated from small-scale pilot studies.

Many simple classical randomized designs satisfy the property that all individuals have an equal marginal probability of treatment; in particular, this includes completely randomized design, which assigns a *p* fraction of individuals uniformly at random from the population to the treatment group. An important property is that neither our estimator nor the appropriate randomized designs need to have knowledge of the underlying network. In fact, all previously proposed solutions required knowledge of the network either for the randomized design or to compute the estimator. In applications where the network is not fully observed, our proposed estimators will still output in an unbiased estimate for the total treatment effect with simple randomizations that can be implemented without knowledge of the underlying network. This provides positive guarantees for settings in which the randomization may be limited due to regulatory policies or lack of precise network information.

A critical assumption that our proposed estimator hinges on is the ability to estimate the baseline parameters. While there may not be sufficient information to estimate individual baselines, the estimators presented in *Corollary 3* require only estimates of the weighted average baseline outcomes, which can be approximated by sampling a small fraction of the population. However, implicit in our assumption is that the average baseline outcomes preexperiment and postexperiment are the same, which excludes settings in which there are time-related dynamics that significantly change the baselines irregardless of the treatment. As an example, suppose that a pharmaceutical company used reported data from state- and national-level public health departments to estimate the average baseline rates of contracting COVID-19 before beginning its clinical trials. The assumption that the baseline outcomes remain fairly constant may be violated if the timescale of the trial period is such that the predominate variant of the virus changes in the interim or the baseline natural immunity level of the population changes significantly.

## Reduction from Network Causal Inference to Estimation of Population Mean

For the remainder of the paper we focus on the following estimator introduced in *Corollary 3*:

$${\hat{\text{TTE}}}_{-\alpha }=\frac{1}{n}\sum_{{}_{i\in [n]}}\frac{Y_{i}(z)-\alpha_{i}}{E[z_{i}]}.$$

It is easy to verify that the bias of the estimator is given by

$$E[{\hat{\text{TTE}}}_{-\alpha }]-\text{TTE}=\frac{1}{n}\sum_{(i,k)\in E}\left(\frac{E[z_{i}]}{E[z_{k}]}-1\right)\gamma_{ik},$$

which is zero when the marginal treatment probabilities are equal across individuals. This estimator is particularly simple because the weights are chosen such that *w_i_* = *v_i_*; i.e., each outcome is incorporated to the estimator with the same weight regardless of whether it is treated or not.

We define the “influence” of individual *i* on the estimate as

$$L_{i}=\beta_{i}+\sum_{k\in [n]}\frac{E[z_{i}]\gamma_{ik}}{E[z_{k}]}.$$

We refer to this as influence because *L_i_* captures the contribution that individual *i* has toward the estimate ${\hat{\text{TTE}}}_{-\alpha }$ when *i* is treated, including the interference effect it has on other individuals as well as the the direct effect it has on itself. *L_i_* does not depend on the realization of the treatment vector.

Under heterogeneous additive network effects,

$${\hat{\text{TTE}}}_{-\alpha }=\frac{1}{n}\sum_{i\in [n]}\left(\frac{\beta_{i}}{E[z_{i}]}+\sum_{k\in [n]}\frac{\gamma_{ik}}{E[z_{k}]}\right)z_{i}≕\frac{1}{n}\sum_{i\in [n]}\frac{L_{i}z_{i}}{E[z_{i}]}.$$

Written in this form, it is clear that the ${\hat{\text{TTE}}}_{-\alpha }$ is simply an inverse propensity-weighted estimator, although the terms *L_i_* are not actually observed. As a result, analytical expressions of the variance of our estimate will follow from direct calculations over the inverse propensity-weighted estimates.

Furthermore, under the sufficient conditions for unbiasedness when $E[z_{i}]=E[z_{k}]$ for all (i,k)∈E, the total treatment effect is equal to the population mean of the influence terms,

$$\mathrm{TTE}=\frac{1}{n}\sum_{i\in [n]}L_{i}.$$

**Fig. 2.**
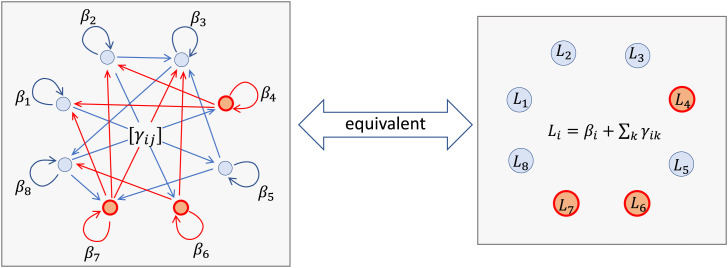
By incorporating baseline estimates, the difficult task of network causal inference reduces to simply estimating a population mean quantity. *(Left)* The TTE under heterogeneous additive network effects is equivalent to the weighted sum of all the edges divided by the total number of individuals. The vertices corresponding to treated individuals are colored red, and all the outgoing edges from treated individuals are colored red. Given prior knowledge of the population average baseline outcomes, the proposed estimator ${\hat{\text{TTE}}}_{-\alpha }$ is equal to the weighted sum of all red edges divided by the number of treated individuals. *(Right)* For each vertex *i*, we can sum the weights of outgoing edges into an influence term *L_i_*. As a result, the task is equivalent to a parallel universe in which each vertex *i* is associated to an influence *L_i_*, and there is no further network interaction. As a result of the proposed experiment, we observe the sum of the influences of all treated individuals. Any randomized design with enough randomness and regularity will be able to guarantee that the average influence of the treated individuals is equal to the population mean influence.

As a result, under the mild assumption of having access to baseline estimates, we have reduced the complex task of network causal inference to a simple task of estimating a population mean of the influence via the sample average of the influence of treated individuals (Fig. 2). This removes all complexity of network interference as the influence terms do not interact. Although our randomized designs and estimators do not require any knowledge of the network, the distribution of the influence will depend on the network and will subsequently affect the variance of our estimator.

## Variance of Proposed Estimator

As there are still many different randomized treatment designs one could use, we compare the variance of our proposed estimator under some commonly used randomized designs. Let *p* denote the treatment budget, such that the size of the treatment group can be at most *p* fraction of the population.

As our estimator corresponds to approximating the total treatment effect with the average of the influence of the treated individuals, this estimator directly inherits properties from the analysis of a sample average estimator for the population mean of the influence. The variance of the estimator is

$$\text{Var}[{\hat{\text{TTE}}}_{-\alpha }]=\sum_{i,j}\frac{L_{i}L_{j}\text{Cov}(z_{i},z_{j})}{n^{2}E[z_{i}]E[z_{j}]}.$$

The randomized design affects the variance through the covariance matrix of the treatment vector.

### Completely Randomized Design.

The completely randomized design generates the treatment assignment vector **z** by selecting a subset of *pn* units to treat uniformly at random out of the size *n* population. This randomized design is commonly used in the classical setting without network interference. Due to the uniform sampling, $E[z_{i}]=p$ for all i∈[n], and

$$\text{Var}[{\hat{\text{TTE}}}_{-\alpha }]=\frac{1-p}{p(n-1)}\text{​}\left(\text{​}\frac{1}{n}\sum_{i\in [n]}L_{i}^{2}-\text{​}{\left(\text{​}\frac{1}{n}\sum_{i\in [n]}L_{i}\text{​}\right)}^{2}\right)\text{​}.$$

The inner expression is equal to the population variance of the influence terms ${\left\{L_{i}\right\}}_{i\in [n]}$, which is bounded by $B^{2}d_{\max}^{2}$, where $d_{\max}$ denotes the maximum outdegree of the network, and *B* is a bound on the direct effect and network effect parameters. A simple bound on the variance thus scales as $B^{2}d_{\max}^{2}/pn$. As a result, the variance will converge to zero for large *n* as long as the number of treated individuals *pn* divided by a constant $B^{2}d_{\max}^{2}$ goes to infinity with large *n*. This is an optimally efficient rate when *B* and $d_{\max}$ are constants, as this requires only the number of treated individuals to be growing larger than a constant as *n* grows. In fact, even optimal estimators for causal effects under SUTVA, without network interference, result in a variance scaling as 1/pn. This means that given the mild assumption of having access to baseline estimates, our approach under fully heterogeneous network effects attains a simple, unbiased, and optimally efficient estimate for the total treatment effect, under the simplest randomized design.

### Cluster-Randomized Design.

The cluster-randomized design partitions the population into clusters, and all individuals in each cluster are either jointly placed in the treatment group or jointly placed in the control group. This is also referred to in the literature as block-randomized design, where blocks refer to clusters. The treatment assignment vector is generated by selecting a subset of *pT* clusters to treat uniformly at random among the *T* clusters. In contrast to completely randomized design, the treatments of individuals within a cluster are perfectly correlated. This randomized design is commonly used in the network interference setting, where the clusters are additionally constructed to minimize edges across clusters, so that an individual and the individual’s local neighbors are jointly assigned to treatment or control as much as possible. In our setting, we do not require such conditions on the construction of the clusters, and thus our clusters may not correspond to tightly connected communities in the network.

The probability that an individual is treated is equal to the probability that the cluster the individual belongs to is treated. Due to the uniform sampling across the clusters, it follows that $E[z_{i}]=p$ for all i∈[n]. Let *T* denote the number of clusters, assuming clusters of uniform size *n*/*T* for simplicity. Let π:[n]→[T] denote the mapping that assigns individuals to clusters. Let $L\prime_{\tau }$ denote the average value of the influence terms within cluster *τ*, $L\prime_{\tau }=\frac{T}{n}\sum_{i:\pi (i)=\tau }L_{i}$. The variance of our estimator under this randomized design is given by

$$\text{Var}[{\hat{\text{TTE}}}_{-\alpha }]=\frac{1-p}{p(T-1)}\text{​}\left(\text{​}\frac{1}{T}\sum_{\tau \in [T]}L\prime_{\tau }{}^{2}-\text{​}{\left(\frac{1}{T}\sum_{\tau \in [T]}L\prime_{\tau }\text{​}\right)}^{2}\right)\text{​}.$$

Observe that the expression is very similar to the variance under the completely randomized design, except we are randomizing over clusters rather than individuals. The inner expression is equal to the variance across clusters of $L\prime_{\tau }$, which is the average influence of individuals in cluster *τ*, bounded by $B^{2}{\left(\max_{\tau \in [T]}{\bar{d}}_{\tau }\right)}^{2}$, where ${\bar{d}}_{\tau }$ denotes the average outdegree of individuals in cluster *τ*. A bound on the variance thus scales as $B^{2}{\left(\max_{\tau \in [T]}{\bar{d}}_{\tau }\right)}^{2}/pT$.

If $d_{\max}$ is constant, and if *T* is asymptotically smaller than *n*, i.e., the size of each cluster is not constant with respect to the population size *n*, then the variance under cluster-randomized design is larger than the variance under completely randomized design. When $d_{\max}$ may be large or even growing with *n*, then the variance might be improved by using cluster-randomized design with an optimal choice of clusters. In particular, there would be a tradeoff between the choice of cluster size and the gain from smoothing out the degree distribution, i.e., the influence, across clusters. In particular, if there is high variation in the influence among individuals, then the variance would be minimized by splitting high-influence individuals across different clusters and grouping them with low-influence individuals, to try to even out the average influence of each cluster. This requires detailed knowledge of the network, however, which is often not available.

### Saturation-Randomized Design.

The saturation-randomized design also assumes that the population is partitioned into clusters, but instead each cluster is treated at a specified saturation level, specifying the percentage of individuals in that cluster that are treated. This is also referred to as a cluster-stratified randomized design. For a cluster *τ*, let $p_{\tau }$ denote the fraction of individuals treated in cluster *τ*, satisfying $\sum_{\tau \in [T]}n_{\tau }p_{\tau }=np$. Let *T* denote the number of clusters, and let π:[n]→[T] map individuals to clusters. Let $n_{\tau }=|i:\pi (i)=\tau |$ denote the size of cluster *τ*. Treatments across different clusters are assigned independently. For each cluster *τ*, a set of $p_{\tau }n_{\tau }$ individuals within the cluster is selected uniformly at random to be treated. Let $V_{\tau }$ denote the variance of the influence terms within cluster *τ*,

$$V_{\tau }=\frac{1}{n_{\tau }}\sum_{i:\pi (i)=\tau }L_{i}^{2}-{\left(\frac{1}{n_{\tau }}\sum_{i:\pi (i)=\tau }L_{i}\right)}^{2}\text{​}.$$

Under uniform saturation, i.e., $p_{\tau }=p$ for all *τ*, the estimator is unbiased as $E[z_{i}]=p$ for all *i*, and the variance is

$$\text{Var}[{\hat{\text{TTE}}}_{-\alpha }]=\frac{1-p}{pn}\sum_{\tau \in [T]}\frac{n_{\tau }^{2}}{n(n_{\tau }-1)}V_{\tau }.$$

This expression essentially scales as $V_{\text{avg}}/pn$, where $V_{\text{avg}}$ denotes the weighted average across cluster variances $V_{\tau }$. In particular, to minimize the variance, each cluster should be chosen to be as homogeneous as possible, so that there is little variation in the influence parameters within each cluster. If $V_{\text{avg}}$ is significantly smaller than the overall population variance over the influence terms ${\left\{L_{i}\right\}}_{i\in [n]}$, then the uniform saturation-randomized design improves in efficiency upon the completely randomized design (CRD). An extreme special case of this randomized design would be the matched-pair randomized design, where the pairs correspond to the clusters, and p=1/2. The pairs are selected to be as similar as possible on known features.

Constructing such clusters requires additional knowledge of covariates or network structure, which is not always available; however, this analysis provides motivation that whenever we do have such auxiliary information at hand, we can only benefit by controlling for the variance that may be related to the auxiliary information. In particular, by grouping similar individuals together, we ensure that the distribution over the auxiliary information in the treated group is as similar as possible to that in the control group.

For a general choice of saturation levels, the estimator may be biased, and the bias will scale proportionally with the sum of the network effect of edges across clusters with different saturation levels. The variance of the estimator is given by

$$\text{Var}[{\hat{\text{TTE}}}_{-\alpha }]=\sum_{\tau \in [T]}\frac{(1-p_{\tau })n_{\tau }^{2}}{p_{\tau }n^{2}(n_{\tau }-1)}V_{\tau }.$$

While varying the saturation levels would introduce bias into the estimator, it may be able to reduce the variance by allocating a larger treated fraction to clusters that are larger or that have larger within-cluster variance $V_{\tau }$. The reduction in variance would need to be carefully balanced with the introduced bias, however, which may be difficult to do since it would require auxiliary information about the cluster variances.

## Conclusion

Estimating the total treatment effect under network interference is an important yet challenging problem. Previous solutions under general models are often too computationally or statistically costly or are limited to very simplistic network structures, inhibiting adoption in practice. As a result, many practical solutions consider strong assumptions on the network effects, which end up reducing the estimation task to a simple regression problem. In contrast, we consider the heterogeneous additive network effects assumption, which imposes additive network effects, but allows for full heterogeneity in the edge-level network effect parameters. This model is significantly more flexible than the simple linear models used in practice. We analyze the properties of individually weighted linear estimators under our model, and our results directly translate into the following insights that are simple to apply in practice: Given baseline estimates, we show that network causal inference is as easy as estimating a population mean! Most notably, our solution does not require knowledge of the underlying network, nor does it critically require strong structural conditions on the network. However, this work does not apply to dynamic settings in which the network or the causal effects change during the course of the experiment.

### Insight 1: Prior Information from Historical Data or Pilot Studies Is Incredibly Valuable; without Such Information, Any Unbiased Estimate Must Use Knowledge of the Network.

We showed that without prior information, there does not exist any unbiased estimate for the total treatment effect that does not use network structure. In particular, we restricted to linear estimators with weights that depend only on whether an individual is treated or not and not on the treatment of the individual’s neighbors. We showed that an unbiased linear estimator exists only if the network can be fully partitioned into disconnected components, such that the randomized design must jointly treat or not treat all individuals in each connected component.

### Insight 2: Use Historical Data or Pilot Studies to Estimate the Population Baseline; Use the Following Simple Unbiased Estimator to Approximate the Total Treatment Effect.

We proposed the following unbiased estimator for any randomized design for which the marginal treatment probability of each individual is *p*,

$${\hat{\text{TTE}}}_{-\alpha }=\frac{1}{p}\left(\frac{1}{n}\sum_{i\in [n]}Y_{i}(z)-\frac{1}{n}\sum_{i\in [n]}\alpha_{i}\right).$$

### Insight 3: The Statistical Properties of the Estimator Depend on the Population Distribution of Individual Influence on the Total Treatment Effect.

Under our additive network effects model, our proposed estimator takes the form of

$${\hat{\text{TTE}}}_{-\alpha }=\frac{1}{n}\sum_{i\in [n]}\frac{L_{i}z_{i}}{E[z_{i}]}\;\text{for}\;L_{i}=\beta_{i}+\sum_{k\in [n]}\frac{E[z_{i}]\gamma_{ik}}{E[z_{k}]},$$

where *L_i_* quantifies individual *i*’s influence on the total treatment effect. This characterization shows that for a sufficiently large population, under simple randomized designs, the distribution of the estimator will also be approximately Gaussian. As a result, variance estimates could be used to design hypothesis tests and compute *P* values.

### Insight 4: Using CRD Results in Optimally Efficient Estimation of the Total Treatment Effect When the Effect Sizes and Outdegrees Are Bounded.

Under CRD, the variance of our estimator is roughly equal to 1/pn times the population variance of the influence terms ${\left\{L_{i}\right\}}_{i\in [n]}$, which is bounded by $B^{2}d_{\max^{2}}$ when the causal effect parameters are bounded by *B* and the outdegree of each individual is bounded by $d_{\max}$. As a result, the estimator is consistent as long as the number of treated individuals *pn* is larger than a constant with respect to the population size *n*.

### Insight 5: Utilize Any Auxiliary Information about Network Structure or Covariates of Individuals in the Population to Control for Variance That May Arise from Heterogeneity among Individuals.

The variance of the estimator is minimized by constructing a randomized design under which the distributions of the influence terms within treated and control are as similar as possible. The influence of individual *i*, denoted by *L_i_*, is a function of the causal effect parameters $\beta_{i},\gamma_{ik}$, but also depends on the network via its local neighborhood structure. If the causal effect parameters or the network structure were related to observed covariates, we could reduce the variance of the estimator by using a uniform saturation-randomized design, where we group together individuals that are similar with respect to observed covariates and local neighborhood structure in the network.

There are many interesting possible extensions for this work, including generalizing the model to add nonlinear terms or relaxing the requirement on having access to estimates of the population baseline, which are being studied by C.L.Y. and coworkers (39, 40) in forthcoming papers. Another interesting direction for future work includes introducing time dependencies in the potential outcomes model and the network.

## Supplementary Material

Supplementary File

## Data Availability

There are no data underlying this work.
