# Supplementary material for: Estimating the total treatment effect in randomized experiments with unknown network structure
Source: Proc Natl Acad Sci U S A. 2022 Oct 24;119(44):e2208975119. doi: 10.1073/pnas.2208975119 (PMC9636977; doi:10.1073/pnas.2208975119)
Supplement: Supplementary File [file pnas.2208975119.sapp.pdf]

PNAS
www.pnas.org

Supplementary Information for

Estimating Total Treatment Effect in Randomized Experiments with Unknown Network Structure

Christina Lee Yu, Edoardo M. Airoldi, Christian Borgs, and Jennifer T. Chayes

Jennifer T. Chayes.
E-mail: jchayes@berkeley.edu

This PDF file includes:

Supplementary text

Supporting Information Text

In this supplementary appendix, we include the proofs of results for estimating the total treatment effect (TTE) stated in the main article. In addition we include extensions of the same approach applied to estimating the average direct treatment effect (ATE) and the average network interference effect (AIE), along with variance calculations for general linear estimators.

Proof of Results on Total Treatment Effect

We restate the results for the total treatment effect here for convenient comparison.

Theorem 1. *Under heterogeneous additive network effects, any unbiased individually weighted linear estimator for total treatment effect must have the form*

$$\widehat{TTE} = \frac{1}{n}\sum_{i\in[n]}\left(\frac{z_i}{\mathbb{E}[z_i]} - \frac{1-z_i}{\mathbb{E}[1-z_i]}\right)Y_i(\mathbf{z}),$$

and the randomized design must satisfy $\mathbb{P}(z_k = z_i) = 1$ for all $(k,i) \in \mathcal{E}$. As a result, there does not exist an unbiased individually weighted linear estimator for the total treatment effect if the network is fully connected.

Proof. Included in main paper. □

Theorem 2. *For any randomized design such that $\frac{\mathbb{P}(z_k=0 \mid z_i=1)}{\mathbb{P}(z_k=1 \mid z_i=0)} = \rho_i$ for all $(k,i) \in \mathcal{E}$ for some values of $\{\rho_i\}_{i\in[n]}$, the following simple estimator*

$$\widehat{TTE}_{-\alpha} = \frac{1}{n}\sum_{i\in[n]}\left(\frac{z_i}{\mathbb{E}[z_i]} - \frac{(1-z_i)\rho_i}{\mathbb{E}[1-z_i]}\right)(Y_i(z) - \alpha_i),$$

produces an unbiased estimate for the total treatment effect under heterogeneous additive network effects.

Proof. After subtracting out the baseline parameters, the constraints for unbiasedness reduce to

- β: for all $i \in [n]$, $w_i\mathbb{E}[z_i] = \frac{1}{n}$,
- γ: for all $(k,i) \in \mathcal{E}$, $w_i\mathbb{E}[z_iz_k] + v_i\mathbb{E}[(1-z_i)z_k] = \frac{1}{n}$.

Satisfying the constraints arising from β and γ results in

$$w_i = \frac{1}{n\mathbb{E}[z_i]} \text{ and } v_i = \frac{\mathbb{E}[z_i(1-z_k)]}{n\mathbb{E}[z_i]\mathbb{E}[(1-z_i)z_k]} \text{ for all } (k,i) \in \mathcal{E}.$$

In order to ensure that such a valid v_i exists, we would need that $\frac{\mathbb{P}(z_k=0 \mid z_i=1)}{\mathbb{P}(z_k=1 \mid z_i=0)} = \rho_i$ for all $(k,i) \in \mathcal{E}$. Under this condition,

$$\frac{\mathbb{E}[z_i(1-z_k)]}{\mathbb{E}[z_i]\mathbb{E}[(1-z_i)z_k]} = \frac{\mathbb{E}[z_i]\mathbb{P}(z_k=0 \mid z_i=1)}{\mathbb{E}[z_i]\mathbb{E}[(1-z_i)]\mathbb{P}(z_k=1 \mid z_i=0)} = \frac{\rho_i}{\mathbb{E}[(1-z_i)]}.$$

□

Corollary 3. *For any randomized design such that $\mathbb{E}[z_i] = \mathbb{E}[z_k]$ for all $(k,i) \in \mathcal{E}$, the following simple estimator*

$$\widehat{TTE}_{-\alpha} = \frac{1}{n}\sum_{i\in[n]}\frac{Y_i(\mathbf{z}) - \alpha_i}{\mathbb{E}[z_i]}$$

produces an unbiased estimate for the total treatment effect under heterogeneous additive network effects. When $\mathbb{E}[z_i] = p$ for all $i \in [n]$, the estimator further simplifies to

$$\widehat{TTE}_{-\alpha} = \frac{1}{p}\left(\frac{1}{n}\sum_{i\in[n]}Y_i(\mathbf{z}) - \frac{1}{n}\sum_{i\in[n]}\alpha_i\right).$$

Proof. First we can verify that if $\mathbb{E}[z_i] = \mathbb{E}[z_k]$, then

$$\frac{\mathbb{P}(z_k=0 \mid z_i=1)}{\mathbb{P}(z_k=1 \mid z_i=0)} = \frac{\mathbb{E}[z_i(1-z_k)]\mathbb{E}[1-z_i]}{\mathbb{E}[z_i]\mathbb{E}[z_k(1-z_i)]} = \frac{(\mathbb{E}[z_i] - \mathbb{E}[z_iz_k])\mathbb{E}[1-z_i]}{\mathbb{E}[z_i](\mathbb{E}[z_k] - \mathbb{E}[z_kz_i])} = \frac{\mathbb{E}[1-z_i]}{\mathbb{E}[z_i]} =: \rho_i.$$

The estimator then results from plugging in the expression for ρ_i into the estimator defined in Theorem 2. When $\mathbb{E}[z_i] = p$, rearranging the expression then results in the simplified form

$$\widehat{\mathrm{TTE}}_{-\alpha} = \frac{1}{p}\left(\frac{1}{n}\sum_{i\in[n]}Y_i(\mathbf{z}) - \frac{1}{n}\sum_{i\in[n]}\alpha_i\right),$$

highlighting that the only knowledge of the baseline parameters needed is the population average baseline. □

Estimating the Average Treatment Effect

The average treatment effect (ATE), also referred to as the direct treatment effect, measures the average difference in outcomes for individuals that is caused only by their own treatments, not including any network effects. It is formally defined as

$$\text{ATE} := \frac{1}{n}\sum_{i\in[n]}(Y_i(\mathbf{e}_i) - Y_i(\mathbf{0})) = \frac{1}{n}\sum_{i\in[n]}\beta_i,$$

where $\mathbf{e}_i$ is the standard basis vector with 1 at component i and zero elsewhere.

Theorem 4. *under heterogeneous additive network effects, any unbiased individually weighted linear estimator for average treatment effect must have the form*

$$\widehat{ATE} = \frac{1}{n}\sum_{i\in[n]}\left(\frac{z_i}{\mathbb{E}[z_i]} - \frac{1-z_i}{\mathbb{E}[1-z_i]}\right)Y_i(\mathbf{z}),$$

and the randomized design must satisfy $z_k \perp\!\!\!\perp z_i$ for all $(k,i) \in \mathcal{E}$.

Proof. Under the heterogeneous additive network effects model, an individually weighted linear estimator takes the value

$$\hat{\text{est}}(\mathbf{w},\mathbf{v}) = \textstyle\sum_{i\in[n]}(w_i z_i + v_i(1-z_i))\alpha_i + \sum_{i\in[n]} w_i z_i \beta_i$$
$$+ \textstyle\sum_{(k,i)\in\mathcal{E}}(w_i z_i + v_i(1-z_i))z_k\gamma_{ki}.$$

This is unbiased for the average treatment effect only if $\mathbb{E}[\hat{\text{est}}(\mathbf{w},\mathbf{v})] = \frac{1}{n}\sum_{i\in[n]}\beta_i$ is satisfied for any configuration of $\{\alpha_i\}_{i\in[n]}, \{\beta_i\}_{i\in[n]}$, and $\{\gamma_{ki}\}_{(k,i)\in\mathcal{E}}$. This requirement results in the following $2n + |\mathcal{E}|$ constraints, which arise from matching coefficients for each of the parameters,

- α: for all $i \in [n]$, $w_i\mathbb{E}[z_i] + v_i\mathbb{E}[1-z_i] = 0$,
- β: for all $i \in [n]$, $w_i\mathbb{E}[z_i] = \frac{1}{n}$,
- γ: for all $(k,i) \in \mathcal{E}$, $w_i\mathbb{E}[z_i z_k] + v_i\mathbb{E}[(1-z_i)z_k] = 0$.

Solving for the weights given the first two constraints results in

$$w_i = \frac{1}{n\mathbb{E}[z_i]} \text{ and } v_i = -\frac{1}{n\mathbb{E}[1-z_i]}.$$

By plugging in these values of w_i and v_i into the third set of constraints arising from γ, it follows that we must satisfy

$$\frac{\mathbb{E}[z_i z_k]}{n\mathbb{E}[z_i]} - \frac{\mathbb{E}[(1-z_i)z_k]}{n\mathbb{E}[1-z_i]} = \frac{\mathbb{P}(z_k=1|z_i=1)}{n} - \frac{\mathbb{P}(z_k=1|z_i=0)}{n} = 0,$$

which implies that $\mathbb{P}(z_k = 1|z_i = 1) = \mathbb{P}(z_k = 1|z_i = 0)$ such that $z_k \perp\!\!\!\perp z_i$ for all $(k,i) \in \mathcal{E}$. ☐

This independence constraint on the randomized design is quite restrictive, yet is easily satisfied by simple Bernoulli randomization. Although many other randomizations may not satisfy independence, if they are "almost" independent, for example in completely randomized design with a large population, then the bias will still be small.

Corollary 5. *Under heterogeneous additive network effects, the Horvitz-Thompson estimator with Bernoulli randomization is an unbiased estimator for the average treatment effect, even when the interference effects are fully dense.*

Proof. This follows from the fact that by definition of Bernoulli randomization, $z_k \perp\!\!\!\perp z_i$ for all $i \neq k$. ☐

Next we consider the scenario when we have estimates of the individual baselines so that we can subtract them from the individual outcomes when constructing our estimator.

Theorem 6. *For any randomized design such that $\mathbb{P}(z_i = 1|z_k = 1) = \rho_i$ for all $(k,i) \in \mathcal{E}$ for some values of $\{\rho_i\}_{i\in[n]}$, the following simple estimator*

$$\widehat{ATE}_{-\alpha} = \frac{1}{n}\sum_{i\in[n]}\left(\frac{z_i}{\mathbb{E}[z_i]} - \frac{(1-z_i)\rho_i}{\mathbb{E}[z_i](1-\rho_i)}\right)(Y_i(z) - \alpha_i),$$

produces an unbiased estimate for the average treatment effect under heterogeneous additive network effects.

The condition that $\mathbb{P}(z_i = 1|z_k = 1) = \rho_i$ for all $(k,i) \in \mathcal{E}$ imposes symmetry in the randomized assignment amongst the neighbors of an individual. Completely randomized design or bernoulli randomization would satisfy this constraint. Alternatively a cluster based randomization would satisfy this constraint if the neighbors of a unit had equal chance of being assigned to the same versus different cluster.

Proof. After subtracting out the baseline parameters, the constraints for unbiasedness reduce to

- β: for all $i \in [n]$, $w_i \mathbb{E}[z_i] = \frac{1}{n}$,
- γ: for all $(k,i) \in \mathcal{E}$, $w_i \mathbb{E}[z_i z_k] + v_i \mathbb{E}[(1-z_i)z_k] = 0$.

Satisfying the constraints arising from β and γ results in

$$w_i = \frac{1}{n\mathbb{E}[z_i]} \text{ and } v_i = -\frac{\mathbb{E}[z_i z_k]}{n\mathbb{E}[z_i]\mathbb{E}[(1-z_i)z_k]} \text{ for all } (k,i) \in \mathcal{E}.$$

In order to ensure that such a valid v_i exists, we would need that there exists values ρ_i such that $\mathbb{P}(z_i = 1 | z_k = 1) = \rho_i$ for all $(k,i) \in \mathcal{E}$. Under this condition,

$$\frac{\mathbb{E}[z_i z_k]}{\mathbb{E}[z_i]\mathbb{E}[(1-z_i)z_k]} = \frac{\rho_i}{\mathbb{E}[z_i](1-\rho_i)}.$$

□

Under completely randomized design with a budget of treating p fraction of the population, $\mathbb{E}[z_i] = p$ and $\rho_i = \frac{pn-1}{n-1}$ for all $i \in [n]$. As a result,

$$\widehat{\text{ATE}}_{-\alpha} = \sum_{i\in[n]} \left(\frac{z_i(n-1) - pn + 1}{(1-p)pn^2} \right) Y_i(z) - \frac{(n-1)}{n(1-p)} \left(\frac{1}{pn} \sum_{i\in[n]} z_i \alpha_i \right) + \frac{pn-1}{(1-p)pn} \left(\frac{1}{n} \sum_{i\in[n]} \alpha_i \right).$$

Furthermore, for large enough n the empirical average baseline of the treated individuals would be similar to the population average baseline, such that this estimator could be computed using the population baseline estimates by

$$\widehat{\text{ATE}}_{-\alpha} \approx \sum_{i\in[n]} \left(\frac{z_i(n-1) - pn + 1}{(1-p)pn^2} \right) Y_i(z) - \frac{1}{pn^2} \sum_{i\in[n]} \alpha_i.$$

Estimation the Average Interference Effect

The average interference effect (AIE), also referred to as the network interference effect, measures the average difference in outcomes of individuals that is caused only due to network effects but not their own direct treatment effects. It is formally defined as

$$\text{AIE} := \frac{1}{n} \sum_{i\in[n]} (Y_i(\mathbf{e}_{[n]\setminus\{i\}}) - Y_i(\mathbf{0})) = \frac{1}{n} \sum_{(k,i)\in\mathcal{E}} \gamma_{ki},$$

where $\mathbf{e}_{\mathcal{S}}$ denotes the n-dimensional vector where $\mathbf{e}_{\mathcal{S}}(x) = 1$ for $x \in \mathcal{S}$ and $\mathbf{e}_{\mathcal{S}}(x) = 0$ for $x \notin \mathcal{S}$.

Theorem 7. *Under heterogeneous additive network effects, there does not exist an unbiased individually weighted linear estimator for the average interference effect.*

Proof. Under the heterogeneous additive network effects model, an individually weighted linear estimator is unbiased for the average interference effect only if $\mathbb{E}[\hat{\text{est}}(\mathbf{w}, \mathbf{v})] = \frac{1}{n} \sum_{(k,i)\in\mathcal{E}} \gamma_{ki}$ is satisfied for any configuration of $\{\alpha_i\}_{i\in[n]}$, $\{\beta_i\}_{i\in[n]}$, and $\{\gamma_{ki}\}_{(k,i)\in\mathcal{E}}$. This requirement results in the following $2n + |\mathcal{E}|$ constraints, which arise from matching coefficients for each of the parameters,

- α: for all $i \in [n]$, $w_i \mathbb{E}[z_i] + v_i \mathbb{E}[1 - z_i] = 0$,
- β: for all $i \in [n]$, $w_i \mathbb{E}[z_i] = 0$,
- γ: for all $(k,i) \in \mathcal{E}$, $w_i \mathbb{E}[z_i z_k] + v_i \mathbb{E}[(1-z_i)z_k] = \frac{1}{n}$.

The first two constraints together require that the weights are all zero, i.e. $w_i = 0$ and $v_i = 0$. However, with zero weights it is impossible to satisfy the constraint arising from γ. □

Next we consider the scenario when we have estimates of the individual baselines so that we can subtract them from the measured outcomes when constructing our estimator.

Theorem 8. *For any randomized design such that $\mathbb{P}(z_k = 1 | z_i = 0) = \rho_i$ for all $(k,i) \in \mathcal{E}$ for some values of $\{\rho_i\}_{i\in[n]}$, the following simple estimator*

$$\widehat{AIE}_{-\alpha} = \frac{1}{n} \sum_{i\in[n]} \frac{(1-z_i)}{\rho_i \mathbb{E}[1-z_i]} (Y_i(z) - \alpha_i),$$

produces an unbiased estimate for the average interference effect under heterogeneous additive network effects.

Proof. After subtracting out the baseline parameters, the constraints for unbiasedness reduce to

- β: for all $i \in [n]$, $w_i \mathbb{E}[z_i] = 0$,

- γ: for all $(k,i) \in \mathcal{E}$, $w_i \mathbb{E}[z_i z_k] + v_i \mathbb{E}[(1-z_i)z_k] = \frac{1}{n}$.

Satisfying the constraints arising from β and γ results in

$$w_i = 0 \text{ and } v_i = \frac{1}{n\mathbb{E}[(1-z_i)z_k]} \text{ for all } (k,i) \in \mathcal{E}.$$

In order to ensure that such a valid v_i exists, we would need that there exists some value ρ_i for which $\mathbb{P}(z_k = 1 | z_i = 0) = \rho_i$ for all $(k,i) \in \mathcal{E}$. Under this condition,

$$\mathbb{E}[(1-z_i)z_k] = \rho_i \mathbb{E}[1 - z_i].$$

□

The condition that $\mathbb{P}(z_k = 1|z_i = 0) = \rho_i$ for all $(k,i) \in \mathcal{E}$ imposes symmetry in the randomized assignment amongst the neighbors of an individual. Completely randomized design or bernoulli randomization would satisfy this constraint. Alternatively a cluster based randomization would satisfy this constraint if the cluster assignments were also randomized, and neighbors of a unit had equal chance of being assigned to the same versus different cluster.

Under completely randomized design with a budget of treating p fraction of the population, $\mathbb{E}[z_i] = p$ and $\rho_i = \frac{pn}{n-1}$ for all $i \in [n]$. Furthermore, for large enough n the empirical average baseline of the treated individuals would be similar to the population average baseline, such that this estimator could be computed using the population baseline estimates by

$$\widehat{\text{AIE}}_{-\alpha} = \frac{n-1}{(1-p)pn^2} \sum_{i \in [n]} (1-z_i) Y_i(z) - \frac{n-1}{(1-p)pn^2} \sum_{i \in [n]} (1-z_i)\alpha_i \approx \frac{n-1}{(1-p)pn^2} \sum_{i \in [n]} (1-z_i) Y_i(z) - \frac{n-1}{pn^2} \sum_{i \in [n]} \alpha_i.$$

Variance for General Linear Estimators

We can compute the variance of an individually weighted linear weighted estimator for commonly used randomizations as a function of the weights w_i and v_i. This could help in choosing the randomization for a given estimator, or choosing the weights to balance between bias and variance for a fixed randomization and estimand. As the expressions given are for a general estimator, this can be used to compute variance for the above presented estimators for the ATE and AIE.

Consider the individually weighted linear weighted estimator of the form

$$\hat{\text{est}}(\mathbf{w}, \mathbf{v}) = \sum_{i \in [n]} (w_i z_i Y_i(\mathbf{z}) + v_i (1 - z_i) Y_i(\mathbf{z})),$$

where w_i and v_i are not functions of the treatment vector $\mathbf{z}$. When we have baseline estimates available, we would instead subtract them from the outcomes when designing the estimator resulting in

$$\hat{\text{est}}_{-\alpha}(\mathbf{w}, \mathbf{v}) = \textstyle\sum_{i \in [n]} (w_i z_i + v_i(1 - z_i)) (Y_i(\mathbf{z}) - \alpha_i).$$

The variance of $\hat{\text{est}}_{-\alpha}(\mathbf{w}, \mathbf{v})$ will in fact follow from our calculations of the variance of $\hat{\text{est}}(\mathbf{w}, \mathbf{v})$ with the simplifying condition that all baseline parameters α_i will be set to zero in the variance calculations since we have already subtracted them from the outcomes in the estimator $\hat{\text{est}}_{-\alpha}(\mathbf{w}, \mathbf{v})$. As such we provide the calculations for the variance of $\hat{\text{est}}(\mathbf{w}, \mathbf{v})$ as this is strictly more general. We define the following expressions

$$L_i = (w_i - v_i)\alpha_i + w_i \beta_i + \sum_{k \in [n]} v_k \gamma_{ik} \mathbb{I}((i,k) \in \mathcal{E})$$

$$H_{ij} = (w_i - v_i)\gamma_{ji} \mathbb{I}((j,i) \in \mathcal{E}) + (w_j - v_j)\gamma_{ij} \mathbb{I}((i,j) \in \mathcal{E}).$$

By expanding the expressions for $Y_i(\mathbf{z})$ from the heterogeneous additive outcomes model and rearranging terms, we can rewrite the estimator in terms of L_i and H_{ij} according to

$$\begin{aligned}
\hat{\text{est}}(\mathbf{w}, \mathbf{v}) &= \sum_{i \in [n]} w_i z_i (\alpha_i + \beta_i + \sum_{k \in [n]} \gamma_{ki} \mathbb{I}((k,i) \in \mathcal{E}) z_k) + \sum_{i \in [n]} v_i (1 - z_i)(\alpha_i + \sum_{k \in [n]} \gamma_{ki} \mathbb{I}((k,i) \in \mathcal{E}) z_k) \\
&= \sum_{i \in [n]} v_i \alpha_i + \sum_{i \in [n]} ((w_i - v_i)\alpha_i + w_i \beta_i) z_i + \sum_{i \in [n]} \sum_{k \in [n]} (w_i - v_i) \gamma_{ki} \mathbb{I}((k,i) \in \mathcal{E}) z_i z_k + \sum_{i \in [n]} v_i (\sum_{k \in [n]} \gamma_{ki} \mathbb{I}((k,i) \in \mathcal{E}) z_k) \\
&= \sum_{i \in [n]} v_i \alpha_i + \sum_{i \in [n]} ((w_i - v_i)\alpha_i + w_i \beta_i + \sum_{k \in [n]} v_k \gamma_{ik} \mathbb{I}((i,k) \in \mathcal{E})) z_i \\
&\quad + \sum_{i < j \in [n]^2} ((w_i - v_i)\gamma_{ji} \mathbb{I}((j,i) \in \mathcal{E}) + (w_j - v_j)\gamma_{ij} \mathbb{I}((i,j) \in \mathcal{E})) z_i z_j \\
&= \sum_{i \in [n]} v_i \alpha_i + \sum_{i \in [n]} L_i z_i + \sum_{i < j \in [n]^2} H_{ij} z_i z_j.
\end{aligned}$$

As a result the variance of $\hat{\text{est}}(\mathbf{w},\mathbf{v})$ is given by

$$\begin{aligned}\text{Var}[\hat{\text{est}}(\mathbf{w},\mathbf{v})] &= \text{Var}[\sum_{i\in[n]} L_i z_i] + 2\text{Cov}[\sum_{i\in[n]} L_i z_i, \sum_{i<j\in[n]^2} H_{ij} z_i z_j] + \text{Var}[\sum_{i<j\in[n]^2} H_{ij} z_i z_j] \\ &= \sum_{i,j\in[n]^2} L_i L_j \text{Cov}[z_i, z_j] + 2\sum_{i\in[n]}\sum_{j<k\in[n]^2} L_i H_{jk}\text{Cov}[z_i, z_j z_k] + \sum_{i<j\in[n]^2}\sum_{k<\ell\in[n]^2} H_{ij}H_{k\ell}\text{Cov}[z_i z_j, z_k z_\ell] \\ &= \sum_{i,j\in[n]^2} L_i L_j \text{Cov}[z_i, z_j] + 2\sum_{i\in[n]}\sum_{j<k\in[n]^2} L_i H_{jk}\text{Cov}[z_i, z_j z_k] + \sum_{i<j\in[n]^2}\sum_{k<\ell\in[n]^2} H_{ij}H_{k\ell}\text{Cov}[z_i z_j, z_k z_\ell] \\ &= \sum_{i,j\in[n]^2} L_i L_j \text{Cov}[z_i, z_j] + 2\sum_{i\in[n]}\sum_{j<k\in[n]^2} L_i H_{jk}\text{Cov}[z_i, z_j z_k] + \sum_{i<j\in[n]^2}\sum_{k<\ell\in[n]^2} H_{ij}H_{k\ell}\text{Cov}[z_i z_j, z_k z_\ell].\end{aligned}$$

For specific randomized designs, we simply plug in the expressions for the moments of the assignment vector $\mathbf{z}$.

Completely Randomized Design. Consider the completely randomized design, which generates the treatment assignment vector $\mathbf{z}$ by selecting a subset of pn units to treat uniformly at random out of the size n population. The second, third, and fourth moments take value:

$$\text{Cov}[z_i, z_j] = \begin{cases} p(1-p) & \text{if } i = j \\ -\frac{p(1-p)}{n-1} & \text{if } i \neq j \end{cases}$$

$$\text{Cov}[z_i, z_j z_k] = \begin{cases} -2p(1-p)(\frac{np-1}{(n-1)(n-2)}) & \text{if } i \notin \{j,k\}, j<k \\ p(1-p)(\frac{np-1}{n-1}) & \text{if } i \in \{j,k\}, j<k \end{cases}$$

$$\text{Cov}[z_i z_j, z_k z_\ell] = \begin{cases} p(\frac{np-1}{n-1})(1-p(\frac{np-1}{n-1})) & \text{if } (i,j)=(k,\ell), i<j \\ p(\frac{np-1}{n-1})(\frac{np-2}{n-2} - p(\frac{np-1}{n-1})) & \text{if } |\{i,j,k,\ell\}| = 3 \\ p(\frac{np-1}{n-1})(\frac{(np-2)(np-3)}{(n-2)(n-3)} - p(\frac{np-1}{n-1})) & \text{if } |\{i,j,k,\ell\}| = 4 \end{cases}$$

The variance calculations follow by plugging the expressions for the moments and rearranging the expression algebraically,

$$\begin{aligned}\text{Var}[\hat{\text{est}}(\mathbf{w},\mathbf{v})] = (1-p)p\frac{n^2}{n-1}\big(\frac{1}{n}\sum_i L_i^2 - \big(\frac{1}{n}\sum_i L_i\big)^2\big) + \frac{(1-p)p(np-1)}{(n-1)(n-2)}\big(n\sum_{j<k\in[n]^2}(L_j+L_k)H_{jk} - 2\sum_i L_i \sum_{j<k\in[n]^2} H_{jk}\big) \\ + p\big(\frac{np-1}{n-1}\big)\big(\big(\frac{np-2}{n-2}\big)\big(\frac{np-3}{n-3}\big) - p\big(\frac{np-1}{n-1}\big)\big)\big(\sum_{i<j\in[n]^2} H_{ij}\big)^2 + \frac{np(1-p)(np-1)(np-2)}{(n-1)(n-2)(n-3)}\sum_{i\in[n]}\big(\sum_{j\neq i\in[n]} H_{ij}\big)^2 \\ + \frac{np(1-p)(np-1)}{(n-1)(n-2)}\big(1 - \frac{np-2}{n-3}\big)\sum_{i<j\in[n]^2} H_{ij}^2.\end{aligned}$$

Cluster RD. Consider the cluster randomized design, which partitions the population into clusters. Each cluster is either fully treated or fully control. The treatment assignment vector is generated by selecting a subset of pT clusters to treat uniformly at random amongst the T clusters.

We can analyze cluster RD using the equations from CRD. Let $z' \in \{0,1\}^T$ denote the cluster treatment vector such that $z_i = z'_{\pi(i)}$. Then the estimator can be written as a sum of z'_τ for $\tau \in T$, in particular

$$\sum_{i\in[n]} L_i z_i = \sum_{i\in[n]} L_i z_i \sum_{\tau\in[T]} \mathbb{I}(\pi(i)=\tau) = \sum_{\tau\in[T]}\left(\sum_{i:\pi(i)=\tau} L_i\right) z'_\tau$$

and

$$\begin{aligned}\sum_{i<j\in[n]^2} H_{ij} z_i z_j &= \sum_{i<j\in[n]^2} H_{ij} z_i z_j \sum_{\tau\leq\tau'\in[T]^2} \mathbb{I}(\pi(i)=\tau, \pi(j)=\tau' \text{ or } \pi(i)=\tau', \pi(j)=\tau) \\ &= \sum_{\tau\in[T]}\left(\sum_{i<j:\pi(i)=\pi(j)=\tau} H_{ij}\right) z'_\tau + \sum_{\tau<\tau'\in[T]^2}\left(\sum_{i<j:\{\pi(i),\pi(j)\}=\{\tau,\tau'\}} H_{ij}\right) z'_\tau z'_{\tau'}.\end{aligned}$$

If we define the notation

$$L'_\tau = \sum_{i:\pi(i)=\tau} L_i \quad \text{and} \quad H'_{\tau\tau'} = \sum_{i<j:\{\pi(i),\pi(j)\}=\{\tau,\tau'\}} H_{ij},$$

it follows that

$$\hat{\text{est}}(\mathbf{w},\mathbf{v}) = \sum_{i\in[n]} v_i\alpha_i + \sum_{\tau\in[T]} (L'_\tau + H'_{\tau\tau})z'_\tau + \sum_{\tau<\tau'\in[T]^2} H'_{\tau\tau'} z'_\tau z'_{\tau'}.$$

We can see that the estimator looks essentially the same as an estimator which operates at the level of the clusters with the modified parameters L' and H'. Therefore, the variance can be computed by substituting the relevant quantities into the variance calculations for completely randomized design.

$$\begin{aligned}
\text{Var}[\hat{\text{est}}(\mathbf{w},\mathbf{v})] &= (1-p)p\frac{T^2}{T-1}\left(\frac{1}{T}\sum_\tau (L'_\tau + H'_{\tau\tau})^2 - \left(\frac{1}{T}\sum_\tau L'_\tau\right)^2\right) \\
&+ \frac{T(1-p)p(Tp-1)}{(T-1)(T-2)} \sum_{\tau<\tau'\in[T]^2} (L'_\tau + H'_{\tau\tau} + L'_{\tau'} + H'_{\tau'\tau'})(H'_{\tau,\tau'} + H'_{\tau',\tau}) \\
&- \frac{2(1-p)p(Tp-1)}{(T-1)(T-2)} \sum_{\tau''}(L'_{\tau''} + H'_{\tau''\tau''}) \sum_{\tau<\tau'\in[T]^2} (H'_{\tau,\tau'} + H'_{\tau',\tau}) \\
&+ p\big(\frac{Tp-1}{T-1}\big)\big(\big(\frac{Tp-2}{T-2}\big)\big(\frac{Tp-3}{T-3}\big) - p\big(\frac{Tp-1}{T-1}\big)\big)\big(\sum_{\tau<\tau'\in[T]^2} (H'_{\tau\tau'} + H'_{\tau'\tau})\big)^2 \\
&+ \frac{Tp(1-p)(Tp-1)(Tp-2)}{(T-1)(T-2)(T-3)} \sum_{\tau\in[T]}\big(\sum_{\tau'\neq\tau\in[T]} (H'_{\tau\tau'} + H'_{\tau'\tau})\big)^2 \\
&+ \frac{Tp(1-p)(Tp-1)}{(T-1)(T-2)}\left(1 - \frac{Tp-2}{T-3}\right) \sum_{\tau<\tau'\in[T]^2} (H'_{\tau\tau'} + H'_{\tau'\tau})^2.
\end{aligned}$$

Cluster stratified RD. Consider the cluster stratified randomized design, which partitions the population into clusters, and within each cluster a specified fraction of the units are treated uniformly at random amongst all units in that cluster. The assignments in different clusters are fully independent. We can rearrange the estimator and use the independence of assignments across different clusters to simplify the variance calculations. Using the definitions of L_i and H_{ij},

$$\hat{\text{est}}(\mathbf{w},\mathbf{v}) = \sum_{i\in[n]} v_i\alpha_i + \sum_{\tau\in[T]}\left(\sum_{i:\pi(i)=\tau} L_i z_i + \sum_{i<j:\pi(i)=\pi(j)=\tau} H_{ij}z_iz_j\right) + \sum_{\tau<\tau'\in[T]^2}\left(\sum_{i<j:\{\pi(i),\pi(j)\}=\{\tau,\tau'\}} H_{ij}z_iz_j\right).$$

Let us define the notation

$$A_\tau = \sum_{i:\pi(i)=\tau} L_iz_i + \sum_{i<j:\pi(i)=\pi(j)=\tau} H_{ij}z_iz_j \quad \text{and} \quad B_{\tau\tau'} = \sum_{i<j:\{\pi(i),\pi(j)\}=\{\tau,\tau'\}} H_{ij}z_iz_j.$$

Because the assignments are independent across different clusters, the variance calculation reduces to

$$\text{Var}[\hat{\text{est}}(\mathbf{w},\mathbf{v})] = \sum_{\tau\in[T]} \text{Var}[A_\tau] + \sum_{\tau\in[T]}\sum_{\tau'\neq\tau\in[T]} \text{Cov}[A_\tau, B_{\tau\tau'}] + \sum_{\tau<\tau'\in[T]^2} \text{Var}[B_{\tau\tau'}] + \sum_\tau \sum_{\tau'\neq\tau'',\tau\notin\{\tau'\tau''\}} \text{Cov}[B_{\tau\tau'}, B_{\tau\tau''}].$$

Observe that $\text{Var}\,[A_\tau]$ is the same as the variance calculations for completely randomized design restricted to the cluster τ, such that

$$\begin{aligned}
\text{Var}\,[A_\tau] &= (1-p_\tau)p_\tau\frac{n_\tau^2}{n_\tau - 1}\big(\frac{1}{n_\tau}\sum_{i:\pi(i)=\tau} L_i^2 - \big(\frac{1}{n_\tau}\sum_{i:\pi(i)=\tau} L_i\big)^2\big) \\
&+ \frac{2(1-p_\tau)p_\tau(n_\tau p_\tau - 1)}{(n_\tau-1)(n_\tau-2)}\big(n_\tau \sum_{i<j:\pi(i)=\pi(j)=\tau} L_iH_{ij} - \sum_{k:\pi(k)=\tau} L_k \sum_{i<j:\pi(i)=\pi(j)=\tau} H_{ij}\big) \\
&+ p_\tau\left(\frac{n_\tau p_\tau - 1}{n_\tau - 1}\right)\big(\big(\frac{n_\tau p_\tau - 2}{n_\tau - 2}\big)\big(\frac{n_\tau p_\tau - 3}{n_\tau - 3}\big) - p_\tau\big(\frac{n_\tau p_\tau - 1}{n_\tau - 1}\big)\big)\big(\sum_{i<j:\pi(i)=\pi(j)=\tau} H_{ij}\big)^2 \\
&+ \frac{n_\tau p_\tau(1-p_\tau)(n_\tau p_\tau - 1)(n_\tau p_\tau - 2)}{(n_\tau-1)(n_\tau-2)(n_\tau-3)} \sum_{i:\pi(i)=\tau}\big(\sum_{j\neq i:\pi(j)=\tau} H_{ij}\big)^2 \\
&+ \frac{n_\tau p_\tau(1-p_\tau)(n_\tau p_\tau - 1)}{(n_\tau-1)(n_\tau-2)}\left(1 - \frac{n_\tau p_\tau - 2}{n_\tau - 3}\right) \sum_{i<j:\pi(i)=\pi(j)=\tau} H_{ij}^2.
\end{aligned}$$

For $\tau \neq \tau'$,

$$\mathrm{Cov}[A_\tau, B_{\tau\tau'}] = \frac{p_\tau(1-p_\tau)p_{\tau'}}{n_\tau - 1}\Big(n_\tau \sum_{i,k:\pi(i)=\tau,\pi(k)-\tau'} L_i H_{ik} - \sum_{i:\pi(i)=\tau} L_i \sum_{h,k:\pi(h)=\tau,\pi(k)=\tau'} H_{hk}$$
$$+ \frac{n_\tau(n_\tau p_\tau - 1)}{n_\tau - 2} \sum_{i<j:\pi(i)=\pi(j)=\tau} \sum_{k:\pi(k)=\tau'} H_{ij}(H_{ik} + H_{jk})$$
$$- \frac{2(n_\tau p_\tau - 1)}{n_\tau - 2} \sum_{i<j:\pi(i)=\pi(j)=\tau} H_{ij} \sum_{h,k:\pi(h)=\tau,\pi(k)=\tau'} H_{hk}$$
$$- \frac{2(n_\tau p_\tau - 1)}{n_\tau - 2} \sum_{i<j:\pi(i)=\pi(j)=\tau} H_{ij} \sum_{h,k:\pi(h)=\tau,\pi(k)=\tau'} H_{hk}\Big).$$

For $\tau \neq \tau'$,

$$\mathrm{Var}[B_{\tau\tau'}] = \frac{n_\tau p_\tau(1-p_\tau)n_{\tau'}p_{\tau'}(1-p_{\tau'})}{(n_\tau - 1)(n_{\tau'} - 1)} \sum_{h,k:\pi(h)=\tau,\pi(k)=\tau'} H_{hk}^2 + \frac{p_\tau p_{\tau'} n_{\tau'}(1-p_{\tau'})(n_\tau p_\tau - 1)}{(n_\tau - 1)(n_{\tau'} - 1)} \sum_{k:\pi(k)=\tau'} \left(\sum_{i:\pi(i)=\tau} H_{ik}\right)^2$$
$$+ \frac{p_\tau p_{\tau'} n_\tau(1-p_\tau)(n_{\tau'}p_{\tau'} - 1)}{(n_\tau - 1)(n_{\tau'} - 1)} \sum_{i:\pi(i)=\tau} \left(\sum_{k:\pi(k)=\tau'} H_{ik}\right)^2$$
$$+ p_\tau p_{\tau'} \left(\left(\frac{n_{\tau'}p_{\tau'} - 1}{n_{\tau'} - 1}\right)\left(\frac{n_\tau p_\tau - 1}{n_\tau - 1}\right) - p_\tau p_{\tau'}\right) \left(\sum_{i,j:\pi(i)=\tau,\pi(j)=\tau'} H_{ij}\right)^2.$$

For a distinct triple $\tau \neq \tau' \neq \tau''$,

$$\mathrm{Cov}[B_{\tau\tau'}, B_{\tau\tau''}] = \frac{p_\tau(1-p_\tau)p_{\tau'}p_{\tau''}}{n_\tau - 1}\Big(n_\tau \sum_{i,k,h:\pi(i)=\tau,\pi(k)=\tau',\pi(h)=\tau''} H_{ik}H_{ih} - \sum_{i,k:\pi(i)=\tau,\pi(k)=\tau'} H_{ik} \sum_{j,h:\pi(j)=\tau,\pi(h)=\tau''} H_{ih}\Big).$$

The final variance of the estimator results from combining these expressions together.
